# The global preclinical research and development landscape for pre-eclampsia and eclampsia therapies

**DOI:** 10.1038/s43856-026-01784-3

**Published:** 2026-07-14

**Authors:** Jessica Postregna, Kate Mills, Fiona Bruinsma, Maya Goldstein, Andrew Tuttle, Lindsay Keir, Cecile Ventola, A. Metin Gülmezoglu, Anne Ammerdorffer, Joshua P. Vogel, Annie R. A. McDougall

**Affiliations:** 1 Women’s, Children’s and Adolescent’s Health Program, Burnet Institute, Melbourne, VIC Australia; 2 School of Public Health and Preventative Medicine, Monash University, Melbourne, VIC Australia; 3 Impact Global Health, Sydney, NSW Australia; 4 Concept Foundation, Geneva, Switzerland

**Keywords:** Reproductive disorders, Drug discovery, Drug development

## Abstract

**Background:**

Surprisingly few medicines are available to prevent or manage pre-eclampsia, all of which are repurposed from non-pregnant populations and conditions. Analyses of clinical research and development pipelines for pre-eclampsia medicines have identified high-potential candidates; however, the preclinical pipeline has not been interrogated. To address this gap, we developed an approach to analyse and rank preclinical candidates and applied it to the preclinical pipeline for pre‑eclampsia and eclampsia.

**Methods:**

We analysed and ranked the potential of 83 preclinical candidates for pre-eclampsia and eclampsia identified in the Maternal Health Pipeline spanning 2000 to 2025. Candidates were assessed across three domains – quality of preclinical evidence, product development stage and implementability. Each question in the prioritisation matrix was assigned a numerical value based on importance. The sum of these values was used to rank the potential of each candidate for future research.

**Results:**

There is a total of 106 candidates in the preclinical pipeline. Twenty-three candidates are excluded from our analysis. Among the 83 candidates analysed, 11 high-potential candidates are identified, and the remaining candidates are ranked as medium (63 candidates) and low potential (nine candidates). Of the candidates analysed, 49.4% are new chemical or biological entities, 37 different binding targets are identified and the proposed mechanism of action for 32 candidates is a reduction in angiogenic factor sFlt-1.

**Conclusions:**

High potential candidates should be prioritised for further development, but progress is restricted by current mechanisms of action and animal models. Increased investment is essential to advance pre-eclampsia therapies into clinical trials.

## Plain Language Summary

Pre-eclampsia is a serious pregnancy condition with few available medicines, most of which were not originally designed for pregnant women. To understand which medicines are being investigated, we aimed to analyse preclinical research on potential drugs, dietary supplements, and biologics for preventing or treating pre‑eclampsia. We developed a structured approach to rank preclinical medicines, evaluating evidence quality, developmental progress, and suitability in clinical practice. Our analysis identified 11 high-potential medicines that warrant further research and development. However, many medicines target the same biological pathway, and evidence relies on animal models that only partially reflect the complexity of the condition in pregnant women. These findings highlight promising medicines but also the need for greater investment to improve preclinical pre-eclampsia development.

## Abstract (web summary)

Postregna et al. evaluate and rank the global preclinical medicines pipeline for pre-eclampsia and develop a prioritisation matrix to assess candidates. Their work identifies 11 high-potential candidates and highlights major gaps limiting the progress of preclinical medicines.

## Introduction

Pre-eclampsia is the most common hypertensive disorder of pregnancy, affecting approximately 4 million women annually worldwide^1^. As a leading cause of maternal and newborn mortality, pre-eclampsia is responsible for more than 60,000 maternal deaths, and approximately 440,000 stillbirths and newborn deaths annually^1–4^. Currently, there are few medicines that can prevent or treat this pregnancy complication - the only cure is delivering the baby and placenta. The lack of effective medicines for pre-eclampsia reflects a more generalised lack of investment in research and development (R&D) for maternal conditions^1^. More recently, the complex molecular mechanisms underlying pre-eclampsia have been elucidated, particularly the impact of elevated placental soluble fms-like tyrosine kinase 1 (sFlt-1). As an anti-angiogenic factor, rising sFlt-1 levels disrupt healthy blood vessel development and cause endothelial dysfunction^5,6^.

Medicines currently recommended by the World Health Organization (WHO) for pre-eclampsia include low-dose aspirin and calcium supplementation for prevention as well as antihypertensives and magnesium sulphate for treatment. However, because most of these medicines are used off-label, their use is associated with several limitations. A recent 2025 Cochrane review found little or no benefit from calcium supplementation, even in populations with low intake^7^. Additionally, these medicines have limited efficacy for some women and remain underused, particularly in low- and middle-income countries (LMICs)^8–10^. This is driven by scarcity of health resources, variable access to healthcare services, and, in the case of preventive low-dose aspirin, the need for risk screening early in pregnancy^11–15^. There have been no advances in new medicines for pre-eclampsia in the last 30 years that have reached clinical practice^16^.

Between 2000 and 2025, 198 candidate medicines for pre-eclampsia and/or eclampsia were identified, across preclinical and clinical research stages. In 2021, through the Accelerating Innovation for Mothers (AIM) project, we mapped the R&D pipeline for pre-eclampsia and eclampsia medicines^17^. We analysed the 32 candidates undergoing clinical research against key criteria in published Target Product Profiles, which have been described previously and adopted by the WHO^17–20^. However, there is currently no defined methodology to assess and rank candidates at the preclinical stage. A critical question remains: which pre-eclampsia medicines from the preclinical R&D pipeline show the greatest potential?

In the current study, we aimed to develop, to the best of our knowledge, a novel approach to ranking preclinical candidates and apply this to the preclinical R&D pipeline for drugs, supplements and biologics to prevent and treat pre-eclampsia and eclampsia. This approach assessed each candidate using three domains - quality of preclinical evidence, the product development stage and implementability. Our assessment has a particular focus on the needs and challenges of pregnant women, providers and healthcare services in LMICs. We also assessed pipeline diversity by mapping candidate binding targets and mechanisms of action. Our analysis identifies 11 high-potential candidates for pre‑eclampsia prevention and treatment. It also highlights important gaps in current preclinical research, emphasising the need for greater investment, further investigation into pre-eclampsia pathophysiology and more clinically relevant research models.

## Methods

### Maternal Health Pipeline database

Previously, as part of the AIM project, we conducted a landscape analysis, mapping the current and historic maternal medicines R&D pipeline. Drugs, dietary supplements and biologics investigated, either in preclinical or clinical settings, for five priority pregnancy-related conditions (pre-eclampsia, preterm birth/labour, postpartum haemorrhage, foetal growth restriction and foetal distress) were included^21^. The methodology to create the database is described elsewhere and the database is publicly available (available at: https://pipeline.impactglobalhealth.org)^21,22^. Briefly, initial candidate identification was sourced by systematically searching Adis Insight, Pharmaprojects, WHO’s International Clinical Trials Registry Platform, PubMed, and relevant grant databases^22^. Preclinical and clinical data gathered were used to complete profiles for each candidate, which were then externally validated by independent clinical research specialists^21^. For this study, we used the 2026 version of the Maternal Health Pipeline.

### Development of prioritisation matrix

There are currently no tools to assess preclinical candidates. To address this gap, we developed, to the best of our knowledge, a novel prioritisation matrix, drawing on several existing tools. Initially, we outlined three overarching domains - evidence quality, product development and implementability. These three domains were chosen based on a literature review, discussion with experts in the AIM project team, and diverse stakeholder feedback from other AIM activities^17–20,23,24^.

**Fig. 1:**
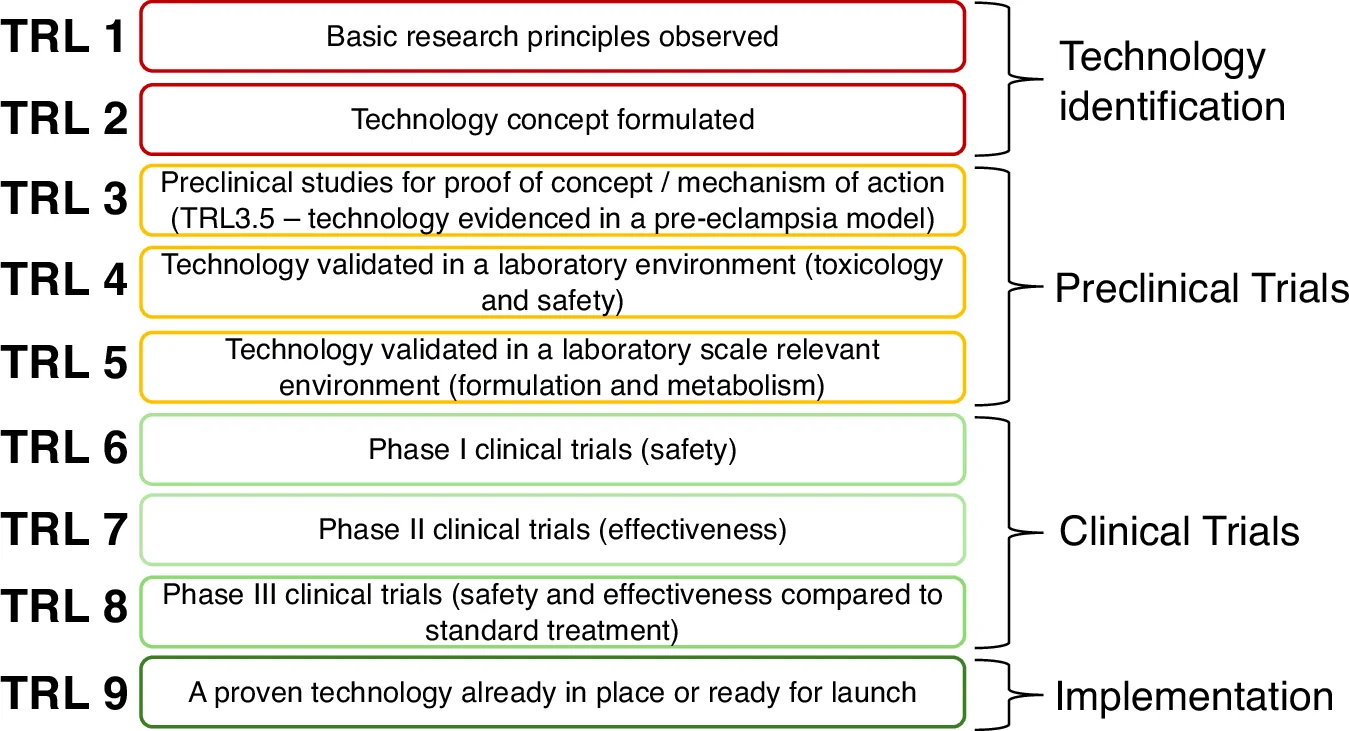
Progression of Technology Readiness Levels (TRLs). TRLs are used to measure the maturity of the development of a product. The levels extend from TRL 1, basic research principles observed, to TRL 9, a proven technology already in place or ready for launch. Generally, preclinical candidates are between TRL 3 and TRL 5.

These domains were then defined using different tools and instruments to inform selected questions. The first domain on quality of preclinical evidence was informed by the Systematic Review Centre for Laboratory Animal Experimentation (SYRCLE) tool and the Quality Assessment Tool for In Vitro Studies (QUIN) tool^25,26^. These tools are designed to assess the quality of preclinical studies for systematic reviews. We used common criteria from each tool to assess both in vitro and in vivo studies using the same questions. For the second domain (product development), we used the established Technology Readiness Levels (TRL), a tool for assessing the maturity of products (Fig. 1)^27–33^. TRLs 1 through 5 are only applicable to preclinical candidates. In this analysis, we have also included TRL 3.5, which was defined as a candidate that had evidence of proof of concept in an appropriate pre-eclampsia model, compared to a TRL 3, which was still in the initial trialling phases of proof of concept in any model. The third domain on implementability was informed by selected criteria in the Target Product Profiles for medicines to prevent and treat pre-eclampsia, namely therapeutic indication, route of administration, cold chain requirements and developer type (pharmaceutical and/or academic)^17,18,20^. A numerical value was assigned to each question’s answer options. Assessment questions with greater importance in transitioning preclinical work into clinical trials, such as reliability of evidence and safety, were given greater weight.

Next, all assessment questions were reviewed by the AIM research team comprised of experts in the maternal health field to ensure content validity. This prioritisation matrix was then piloted on 10 candidates from the Maternal Health Pipeline to further refine the questions according to information available for each candidate. The prioritisation matrix, containing the complete list of questions used to assess potential, is described in Supplementary Table 1.

### Preclinical maternal health pipeline diversity

The diversity of the pipeline was assessed by mapping the research trends over the time period 2000-2025, as well as the binding targets and mechanisms of action of candidates. Information on drug-specific binding sites was collected from information in the pipeline, supplemented with further literature searches.

### Analysis of preclinical candidates

First, we considered all preclinical candidates in the R&D pipeline, and excluded those where: (1) no primary preclinical data were identified, and only a hypothesised role in prevention or treatment of pre-eclampsia was presented in the literature; (2) data was not publicly available or preclinical data was only available from published abstracts; or (3) preclinical evidence suggested significant adverse effects, such as foetal loss or reabsorption. NCE candidates that have progressed to the clinical phase of development, but trials are not yet completed, will not be analysed using the prioritisation matrix but described descriptively using information in the pipeline, supplemented with further grey literature searches.

The remaining candidates were included and analysed using the prioritisation matrix. Two reviewers scored each candidate independently based on the pre-defined criteria in the prioritisation matrix (Supplementary Table 1, Supplementary Data 1). Any differences were resolved via discussion or consulting a third reviewer. Product development and implementability were assessed overall for each candidate, whereas each preclinical study for a single candidate was assessed separately for quality of evidence. For candidates with more than one published study, the average score was calculated and adjusted for the total number of studies (Supplementary Table 1). Each candidate received a final score out of 90 with the following contributions: quality of evidence (58.9%; max total score of 53), product development (28.9%; max total score of 26) and implementability (12.2%; max total score of 11). Once the final score was assigned to each candidate, they were ranked as high, medium or low potential based on cut-off scores (Supplementary Table 1).

### Statistics and reproducibility

As this study was a descriptive evaluation and scoring of existing published data, inferential statistics, sample size and experimental replicates were not applicable. Two reviewers independently assessed each candidate using predefined criteria, with discrepancies resolved by discussion. All data were extracted from publicly accessible publications and databases to ensure reproducibility.

## Results

### Characteristics of the preclinical medicines pipeline for pre-eclampsia

The Maternal Health Pipeline database included 106 preclinical candidate medicines for the prevention and treatment of pre-eclampsia and eclampsia. Following the exclusion of 23 candidates (Supplementary Table 2), 83 candidates were analysed. Of these, 26 (31.3%) were being actively investigated and 57 (68.7%) were inactive (i.e., no published activity since 2023; Table 1). In total, 45 (54.2%) candidates were classified as drugs, 20 (24.1%) as biologics and 18 (21.7%) as dietary supplements (Table 1). Forty-one (49.4%) candidates were new chemical or biological entities (NCEs), while the remaining 42 (50.6%) candidates were repurposed (Table 1). Seven (8.4%) targeted preterm pre-eclampsia, two (2.4%) targeted term pre-eclampsia, two (2.4 %) targeted both preterm and term pre-eclampsia, while 72 (86.8%) were undefined (Table 1). Five (6.0%) candidates are being investigated for pre-eclampsia prevention, 50 (60.2%) candidates for pre-eclampsia treatment and one (1.2%) candidate for pre-eclampsia and eclampsia treatment (Table 1). Fourteen (16.9%) candidates were being explored as both a prevention and treatment for pre-eclampsia and 13 (15.7%) candidates were only investigated for symptom management for pre-eclampsia (Table 1). Sixty-six (79.5%) candidates were being investigated by academic institutes, six (7.2%) by pharmaceutical companies and 11 (13.3%) candidates were being explored independently by both academic and pharmaceutical enterprises (Table 1). Preclinical studies for these candidates included in vitro (human placental cell) models and/or in vivo (animal) models. The most common models of pre-eclampsia were the N(ω)-nitro-L-arginine methyl ester (L-NAME) pregnant rat model (36 studies), the reduced uteroplacental perfusion pressure (RUPP) pregnant rat model (13 studies) and the lipopolysaccharide (LPS) pregnant rat model (12 studies).

**Table 1:** Summary characteristics of the preclinical Maternal Health Pipeline for pre-eclampsia

	Pre-eclampsia prevention (*n* = 5)	Pre-eclampsia treatment (*n* = 51)	Pre-eclampsia prevention and treatment (*n* = 14)	Pre-eclampsia symptom management (*n* = 13)	TOTAL (*n* = 83)
STATUS					
Active	1(1.2%)	20(24.1%)	3(3.6%)	2(2.4%)	26(31.3%)
Inactive	4(4.8%)	31(37.3%)	11(13.3%)	11(13.3%)	57(68.7%)
CLASSIFICATION					
Drugs	3(3.6%)	35(42.2%)	3(3.6%)	4(4.8%)	45(54.2%)
Dietary supplements	1(1.2%)	5(6.0%)	6(7.2%)	6(7.2%)	18(21.7%)
Biologics	1(1.2%)	11(13.3%)	5(6.0%)	3(3.6%)	20(24.1%)
ARCHETYPE					
NCE	4(4.8%)	24(28.9%)	7(8.4%)	6(7.2%)	41(49.4%)
Repurposed	1(1.2%)	27(32.5%)	7(8.4%)	7(8.4%)	42(50.6%)
ONSET					
Preterm pre-eclampsia	-	6(7.2%)	-	1(1.2%)	7(8.4%)
Term pre-eclampsia	1(1.2%)	-	-	-	1(1.2%)
Preterm and term pre-eclampsia	-	2(2.4%)	-	-	2(2.4%)
Undefined	4(4.8%)	43(51.8%)	14(16.9%)	12(14.5%)	73(88.0%)
INVESTIGATOR					
Academic	4(4.8%)	42(50.6%)	10(12.0%)	10(12.0%)	66(79.5%)
Pharmaceutical	1(1.2%)	3(3.6%)	1(1.2%)	1(1.2%)	6(7.2%)
Both	-	6(7.2%)	3(3.6%)	2(2.4%)	11(13.3%)

**Fig. 2:**
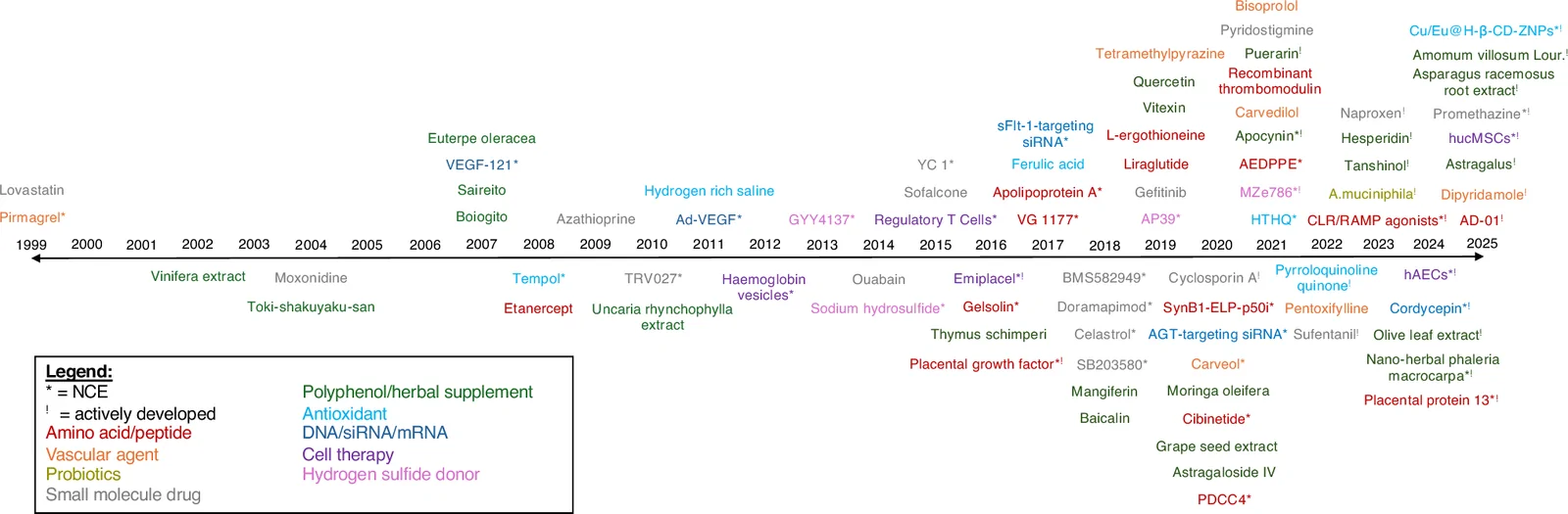
Timeline of preclinical candidate medicines for pre-eclampsia. Each candidate appears in order of the publication date of the first preclinical study investigating its use for pre-eclampsia from 2000 to 2025. Asterisks denote new chemical or biological entities (NCEs), and a circle represents a candidate that is currently in active development. Candidate medicines are colour coordinated based on drug classification.

The number of preclinical candidate medicines increased over the 10-year period from 2015–2025 (Fig. 2). Preclinical studies of herbal supplements and small molecule drugs were published across the 20-year period, though hydrogen sulphide donors emerged in later years. With the development of biologic technologies, pre-eclampsia-specific DNA, RNA and cell therapies have also been introduced into the R&D pipeline since 2012.

**Fig. 3:**
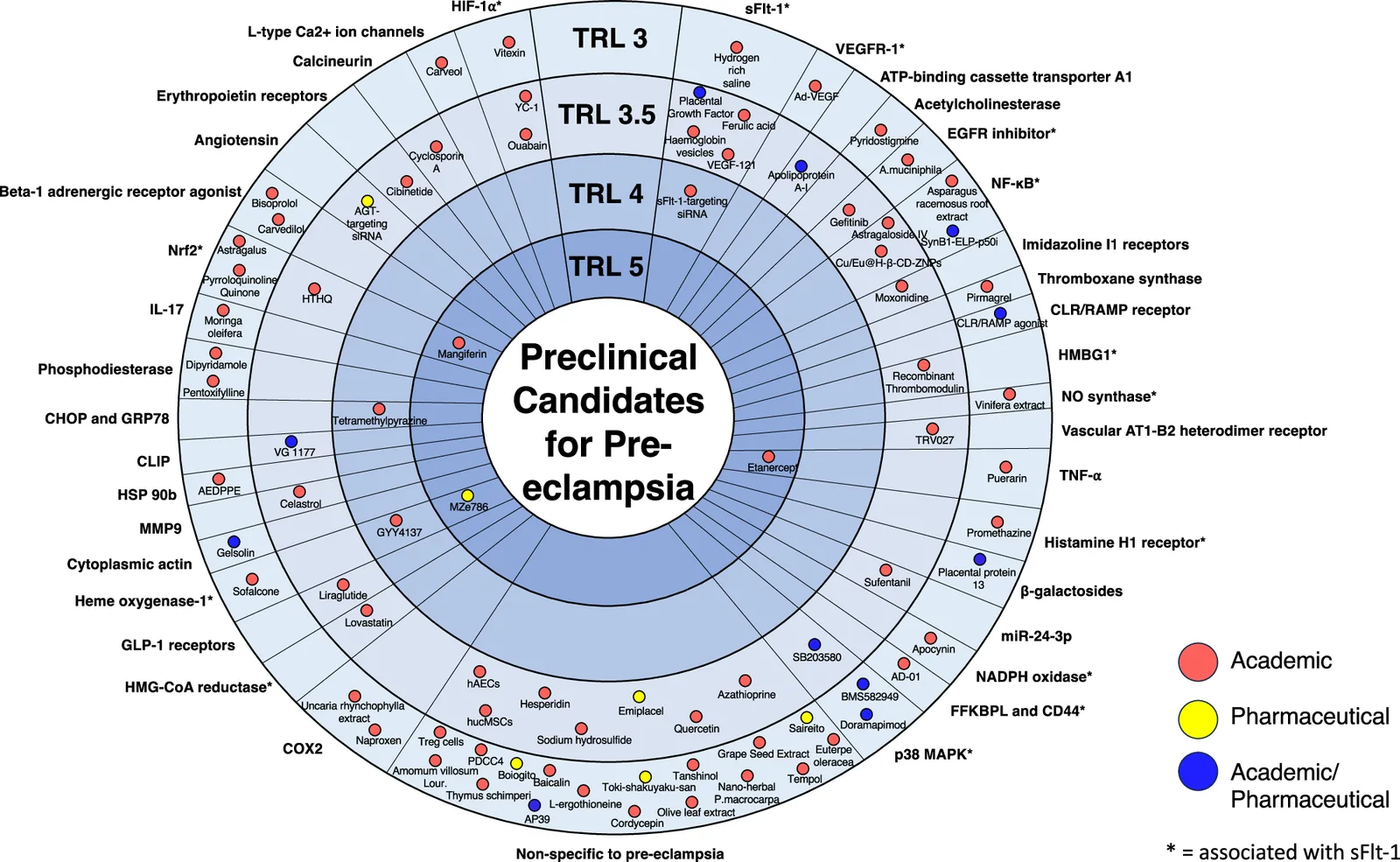
Bullseye diagram of preclinical candidates for pre-eclampsia. Each ring represents a different technology readiness level (TRL), where the outermost ring is a TRL 3 and the innermost a TRL 5. The diagram is split into the different target binding sites for each candidate. The developer of each candidate is divided into academic (red), pharmaceutical (yellow) or academic/pharmaceutical (blue) enterprises. Asterisks denote a binding target related to the sFlt-1 signalling pathway.

Across the 83 candidates, 37 different binding targets were identified. The most common targets were sFlt-1 (six candidates, 7.2%), nuclear factor-κB (NF-κB; four candidates, 4.8%), nuclear factor erythroid 2-related factor 2 (Nrf-2; four candidates, 4.8%), p38 mitogen-activated protein kinases (MAPK; three candidates, 3.6%), haem-oxygenase-1 (HO-1; three candidates, 3.6%), hypoxia-inducible factor 1α (HIF-1α; three candidates, 3.6%), beta-1 adrenergic receptor (two candidates, 2.4%), cyclooxygenase-2 (COX-2; two candidates, 2.4%), epidermal growth factor receptor (EGFR; two candidates, 2.4%), phosphodiesterase (PDE; two candidates, 2.4%) and tumour necrosis factor-alpha (TNF-α; two candidates, 2.4%) (Supplementary Data 2). All other candidates targeted one specific binding site (26 candidates, 31.3%). For 24 candidates (28.9%) no specific target binding site was identified (Fig. 3, Supplementary Data 2). Despite differences in binding sites, many candidate medicines targeted similar mechanisms of action or signalling pathways (32 candidates; 38.6%). These pathways aimed to decrease levels of sFlt-1 or inhibit its release via target binding sites for coenzyme A reductase, EGFR, FK506-binding protein like (FKBPL), HO-1, HIF-1α, high mobility group box 1 (HMGB1), histamine H1 receptor, HO-1, NADPH oxidase (NOX), NF-κB, nitric oxide synthase, Nrf-2, p38 MAPK and vascular endothelial growth factor receptor (VEGFR-1) to activate these signalling pathways.

### Potential of preclinical candidate medicines for pre-eclampsia

Of the 83 candidates analysed, the majority were at TRL 3 (47 candidates), followed by TRL 3.5 (29 candidates). Only four candidates were at TRL 4 and three candidates at TRL 5. Using the prioritisation matrix, 11 candidates were ranked as high potential, 63 as medium potential and nine as low potential (Supplementary Data 1). The quality of evidence across preclinical studies, generally, had a moderate risk of bias, where very few studies reported the use of randomisation or blinding during experimentation. For the majority of candidates, a mechanism of action was reported. The proposed mechanism of action was related to pre-eclampsia in 67.5% (56 candidates). Most of these candidates (38; 52.8%) reported evidence supporting the proposed mechanism of action from a pre-eclampsia model. Despite a safe human dose being established in 43 (51.8%) candidates, 20 were using an unsafe human dose in preclinical experiments (AD-01, Amomum villosum Lour., asparagus racemosus root extract, astragalus, bisoprolol, dipyridamole, hesperidin, liraglutide, moxonidine, olive leaf extract, pentoxifylline, promethazine, pyridostigmine, pyrroloquinoline quinone, saireito, *scutellaria baicalensis* root extract (baicalin), tetramethylpyrazine, thymus schimperi, toki-shakuyaku-san and vinifera extract)^34–58^. Overall, half of all candidate medicines (46; 55.4%) could be administered in oral formulations; the remaining were proposed to be administered as intraarterial, intravenous, subcutaneous, intraperitoneal or intramuscular injections. For two candidates, an intended route of administration was not stated. Just over half of all candidates (43; 51.8%) would require cold chain transport and storage.

#### High potential candidates

Eleven candidates were identified as high potential (Table 2) – gefitinib, MZe786, cyclosporin A, SynB1-ELP-p50i, puerarin, mangiferin, recombinant human placental growth factor (rhPlGF), l-ergothioneine, azathioprine, etanercept and sufentanil. Eight (72.7%) were investigated by academic institutions and one (9.1%) by a pharmaceutical company. The remaining two (18.2%) candidates are being explored by both, independently. Seven high potential candidates were being investigated for pre-eclampsia treatment, one for pre-eclampsia prevention and three for both prevention and treatment. While most candidates utilise an oral route of administration (gefitinib, MZe786, cyclosporin A, puerarin, mangiferin, l-ergothioneine, and azathioprine), some required subcutaneous (etanercept and rhPlGF) or intravenous injections (SynB1-ELP-p50i and sufentanil). Cold chain storage and transportation were required for six candidates (SynB1-ELP-p50i, puerarin, mangiferin, rhPlGF, l-ergothioneine and etanercept). Gefitinib was the only high-potential candidate with a known safety concern in pregnancy. Alongside sFlt-1 inhibition, gefitinib also inhibits EGFR, which is involved in an essential signalling pathway for normal placental development^59^. As such, regulatory bodies like the Therapeutic Goods Administration have classified it as Category C (suspected of causing harmful effects on the human foetus or neonate without causing malformations) based on other animal studies; no human trials have measured its impact on foetal safety^60,61^. However, no adverse effects were observed in the gefitinib study included in the Maternal Health Pipeline^59^.

**Table 2:** Summary of high potential preclinical candidate medicines for pre-eclampsia/eclampsia

	Preclinical Candidate	Development Status	Archetype	Target	Investigator	Therapeutic Indication	Quality	Product Development	Implementability	Final Score
HIGH POTENTIAL CANDIDATES										
1	MZe786^62,63^	Active	NCE	HO1/Hmox1 promoter	Pharmaceutical	Prevention	34	20	9	63
2	Gefitinib^59^	Inactive	Repurposed	EGFR inhibitor	Academic	Treatment	37	16	10	63
3	Cyclosporin A^65^	Active	Repurposed	Calcineurin	Academic	Treatment	35	15	10	60
4	SynB1-ELP-p50i^66^	Inactive	NCE	NF-κB	Pharmaceutical/Academic	Treatment	39	16	3	58
5	Puerarin^67,68^	Active	Repurposed	TNF-α	Academic	Prevention/ Treatment	34	16	7	57
6	Mangiferin^69,70^	Inactive	Repurposed	PI3K/Akt/mTOR signalling pathway	Academic	Prevention/Treatment	31	19	7	57
7	rhPlGF^71–73^	Active	NCE	sFlt-1	Pharmaceutical/Academic	Treatment	30.33	21	4	55.33
8	L-ergothioneine ^75^	Inactive	Repurposed	Non-specific	Academic	Prevention/ Treatment	31	17	7	55
9	Azathioprine^76^	Inactive	Repurposed	Non-specific	Academic	Treatment	27	18	10	55
10	Etanercept^77,78^	Inactive	Repurposed	TNF-α	Academic	Treatment	30	20	5	55
11	Sufentanil^79^	Active	Repurposed	miR-24-3p	Academic	Treatment	33	15	7	55

MZe786 is a hydrogen sulphide-releasing aspirin that targets the HO-1 promoter, which is consequently stimulated as a negative regulator of sFlt-1 (Table 2)^62^. MZe786 is an NCE actively in development by MirZyme Therapeutics (United Kingdom) for the prevention of pre-eclampsia. Findings from two preclinical studies in two different animal models (RUPP mouse model and HO-1 haploid-deficient pregnant mouse model in a high sFlt-1 environment) found that MZe786 was able to successfully prevent pre-eclampsia by reducing mean arterial pressure (MAP), glomerular damage, and levels of sFlt-1 alongside reducing foetal loss and foetal growth restriction^62,63^. As the next step, MirZyme Therapeutics is currently raising funds to advance this candidate into clinical trials^64^.

Gefitinib is a repurposed drug originally developed to treat cancer^21^. It was being investigated in Australia for pre-eclampsia treatment by blocking EGFR; however, it is now inactive^59^. One preclinical study reported a reduction in sFlt-1 following gefitinib treatment in both in vitro (preterm preeclamptic placental tissue and primary cytotrophoblasts, isolated from term normotensive placental tissue) and a pregnant mice model, without affecting pup or placental weight^59^. Gefitinib was administered using tail-vein injection, though it can also be taken orally.

Cyclosporin A is an inhibitory repurposed drug that targets calcineurin to suppress immune responses by blocking the activation and proliferation of cytotoxic T-cells^21^. Academic institutes in China are actively investigating it as a treatment for preeclampsia and eclampsia. One preclinical study involving administration of cyclosporin A to lipopolysaccharide-induced rats reported significantly reduced mean systolic blood pressure, proteinuria, serum IL-1β, TNF-α, and IL-17 levels, all while increasing foetal and placental weight^65^. Treatment with cyclosporin A also decreased the seizure severity and prolonged the latency to seizure^65^. Although this study was performed using a once-off intravenous infusion of cyclosporin A, in humans it can be taken orally.

SynB1-ELP-p50i is a polypeptide delivery system specifically designed to inhibit the NF-κB signalling pathway and decrease TNF-α levels (Table 2)^21^. Developed by Leflore Technologies (based in USA), SynB1-ELP-p50i was an NCE being investigated for pre-eclampsia treatment but is now inactive. Human umbilical vein endothelial cells that were stimulated with TNF-α showed that SynB1-ELP-p50i blocked the translocation of NF-κB into the nucleus^66^. When administered in a RUPP pregnant rat model, pre-eclampsia outcomes were improved by ameliorating placental ischaemia-induced hypertension and reducing placental TNF with no signs of toxicity^66^.

Puerarin is a repurposed dietary supplement that is proposed to help prevent and treat pre-eclampsia by inhibiting TNF-α. Actively developed by Southern Medical University and the University of Science and Technology, both in China, puerarin exerts anti-apoptosis and anti-inflammatory effects^67^. Two preclinical studies have been performed; one in the LPS rat and the other in the L-NAME rat^67,68^. The first study found that puerarin ameliorated the progression of pre-eclampsia by associated inflammation and apoptosis^67^. The other study found that puerarin alleviated hypertension and proteinuria while protecting from oxidative stress and correcting gut dysbiosis^68^.

Mangiferin is a repurposed dietary supplement - a natural, non-steroidal polyphenol^21^. Now inactive, academic researchers in China were investigating mangiferin for the prevention and treatment of pre-eclampsia by targeting upregulation of Nrf-2 via the Pl3K/Akt/mTOR signalling pathway. Mangiferin has high antioxidant, anti-inflammatory and anticancer effects^69,70^. Two preclinical studies have been conducted in two different models: the hyperuricemic rat and the phosphatidylserine/dioleoyl-phosphatidylcholine mouse^69,70^. Both studies found that mangiferin was able to reduce systolic blood pressure and increase nitric oxide secretion as well as attenuate sFlt-1 and rescue placental growth factor expression in the blood and placenta alongside the increased foetal weight in pre-eclamptic models.

rhPlGF protein is an NCE, biological candidate that targets sFlt-1, which is being actively developed by Aggamin LLC (in the USA) and multiple international academic affiliates for pre-eclampsia treatment^21^. The first study in a RUPP pregnant rat model found that intraperitoneal injections of rhPlGF reduced MAP, oxidative stress and sFlt-1 levels while increasing glomerular filtration^71^. The second study utilised uterine artery ligation in a non-human pregnant primate (*Papio hamadryas* - hamadryas baboon) model, and reported that systolic blood pressure, proteinuria and uteroplacental ischaemia were rescued in response to subcutaneous rhPlGF over a five-day period of injections^72^. An ex vivo study in plasma and placenta samples from normotensive and pre-eclamptic patients showed that binding placental growth factor to excess sFlt-1 is a viable treatment for pre-eclampsia^73^.

L-ergothioneine is an amino acid and repurposed dietary supplement hypothesised to reduce mitochondrial reactive oxygen species production^74,75^. Although the specific binding partner in pre-eclampsia is not clear, l-ergothioneine is known to bind to Organic Cation Transporter 1 to facilitate antioxidant effects^21,74^. L-ergothioneine was being explored for the prevention and treatment of pre-eclampsia by multiple academic institutions, but is now inactive. One preclinical study in the RUPP pregnant rat model reported that mitochondrial reactive oxygen species production mediates the mitochondrial dysfunction demonstrated in pre-eclampsia, leading to oxidative damage and a poorly perfused placenta^75^. This study used l-ergothioneine to restore MAP and improve pre-eclampsia outcomes by significantly increasing pup weight while decreasing sFlt-1 levels^75^.

Azathioprine is a repurposed drug that was being investigated by Scott and White Memorial Hospital in the USA but is currently inactive (Table 2)^21^. While no mechanism of action specific to pre-eclampsia has been proposed, azathioprine is known to have multiple binding partners, including hypoxanthine-guanine phosphoribosyl transferase and thiopurine methyltransferase enzymes^21^. One study using a deoxycorticosterone acetate salt-low renin pregnant rat model reported oral azathioprine significantly attenuated hypertension, proteinuria and endothelial dysfunction in this model^76^.

Etanercept is an inactive biologic agent that is FDA-approved for use in autoimmune diseases such as rheumatoid arthritis^21^. The University of Mississippi (USA) was repurposing etanercept for treatment of pre-eclampsia through the inhibition of TNF-α^77,78^. Two preclinical studies were performed; both were investigated in the RUPP rat model^77,78^. Both studies reported a decrease in TNF-α, which lowered the cytolytic natural killer and subsequently improved MAP^77,78^. Importantly, etanercept was able to improve the characteristics of pre-eclampsia without worsening foetal outcomes^77,78^.

Sufentanil is currently being investigated by the Maternity and Child Health Hospital in China for the treatment of pre-eclampsia^21^. It is a repurposed drug that binds to microRNA-24-3p (miR-24-3p)^79^. One preclinical study has been conducted in pre-eclamptic placental samples and the L-NAME rat model^79^. Sufentanil boosted cell proliferation in vitro through the downregulation of miR-24-3p. In vivo results showed that treatment with sufentanil reduced systolic blood pressure and proteinuria while increasing foetal weight^79^.

#### Medium and low potential candidates

Of the 83 candidates, 63 were medium potential and nine were low potential candidates. Some medium potential candidates have evidence of efficacy and mechanism of action from preclinical studies and were at TRL 4 (GYY4137, pyridostigmine, sFlt-1-targeting small interfering RNA (siRNA) and tetramethylpyrazine; Supplementary Data 2)^39,40,57,80–84^. However, most medium-potential candidates, including some at TRL 4, generally had invasive routes of administration involving intravenous, subcutaneous or intramuscular injections. Additionally, most of these candidates require cold chain to be transported and stored stably (Supplementary Data 1). Full results for medium and low potential candidates are included as supplementary text (Supplementary Text 1).

### NCE candidate medicines planned for clinical trials

There are seven NCE candidates that are planned or have not yet completed clinical trials for pre-eclampsia (Table 3). Five were identified using the Maternal Health Pipeline and an additional two were identified from a grey literature search of developer websites and press releases. Five of these candidates are being investigated for pre-eclampsia treatment, while tafoxiparin is being investigated for both prevention and treatment and S-Nitrosoglutathione for symptom management (Table 3). Two of these candidates, RMC 035 and S-Nitrosoglutathione, are currently inactive. More recently, RMC 035 and tafoxiparin have been investigated for alternative indications, the prevention of acute kidney injury and cervical ripening respectively^85,86^.

**Table 3:** Details of NCE candidates for pre-eclampsia that have planned or not yet completed clinical trials

NCE candidate	Investigator	Therapeutic indication	Description	Trial status	Development status
CBP-4888^88^	Comanche Biopharma	Treatment	sFlt-1-targeting siRNA	Phase I – currently recruiting women with preterm pre-eclampsia in Australia^88^	Active
DM199^89^	DiaMedica Therapeutics	Treatment	Protein replacement therapy to restore levels of KLK1	Phase II – recruiting women with pre-eclampsia and/or foetal growth restriction in South Africa^89^	Active
*MOm303^107^	Beech Biotech	Treatment	“Mother-only” monoclonal antibody that binds and eliminates maternal sFlt-1	Preparing for clinical trials	Active
RMC 035^108^	A1M Pharma	Treatment	Recombinant human alpha-1-microglobulin removes free radicals and alleviates oxidative stress	Phase Ia completed in healthy participants, no development reported for pre-eclampsia since^108^	Inactive
*sFlt-1 asialoglycoprotein receptor targeting chimeras (ATACs)^109^	Avilar Therapeutics	Treatment	Elimination of sFlt-1 using the natural asialoglycoprotein receptor degradation pathway	Preparing for clinical trials	Active
S-Nitrosoglutathione^110^	Cambridge University Hospitals NHS Foundation Trust	Symptom management	Improvement of arterial stiffness via downregulation of S-nitrosylated proteins	Phase I completed in 2014 in women with early onset pre-eclampsia in the United Kingdom^110^, no development reported for pre-eclampsia since	Inactive
Tafoxiparin^111^	Dilafor AB	Prevention/ Treatment	Heparan sulphate mimetic without anticoagulant activity	Phase II ended prematurely due to slow recruitment (Italy)^111^	Active

Asterisks (*) denote candidates that were identified through grey literature search. These candidates are not in the Maternal Health Pipeline as the methodology relies on published clinical trial databases and peer-reviewed literature.

## Discussion

The preclinical R&D pipeline for pre-eclampsia medicines uncovers great innovation potential, especially when compared to the clinical pipeline, which is dominated by repurposed medicines^17^. Approximately 50% of candidates in preclinical development were NCEs that target a range of binding partners. Increasing innovation of NCEs within the preclinical pipeline, such as molecular medicines (RNA/DNA or protein-based therapies), means that developing candidates are more targeted and specific to the complexity of pre-eclampsia and its associated complications^87^. Notable examples of NCEs in clinical development for pre‑eclampsia treatment include CBP‑4888 (Comanche Biopharma, Phase I), DM199 (DiaMedica Therapeutics, Phase II), MOm303 (Beech Biotech, planned for clinical trials), and sFlt-1 ATACs (Avilar Therapeutics, planned for clinical trials)^21,64,88,89^. Collectively, these candidates demonstrate the translational potential of purpose‑designed, mechanism‑based therapies, with several already in clinical trials and others advancing towards clinical investigation. However, 90% of all clinical drug development fails, largely because of reproducibility issues and difficulty translating preclinical evidence to human applications^90,91^. Due to this high rate of attrition, it is critical that multiple, diverse candidates continue to be developed so that even one medicine can reach the market. Regulatory challenges in preeclampsia drug development is further hindered by limited industry engagement, widespread off‑label use of existing medications, and hesitancy to conduct clinical trials in pregnant populations^92,93^. Product development should not be limited to medicines, as emerging research suggests that interventional procedures, including apheresis, may also offer therapeutic benefit for pre-eclampsia management^94,95^. Despite the diversity of the pipeline initially appearing high, our analysis highlights that most candidates are targeting the same mechanism of action, namely a reduction in sFlt-1 signalling. Although the majority of global morbidity and mortality for pre-eclampsia occurs in LMICs, the development of high-potential candidate medicines remains concentrated in high-income countries^2^. Here, we have identified eleven preclinical candidates with the strongest potential, warranting investment and advancement to next-stage clinical research.

Few studies have analysed the global preclinical R&D medicines pipeline for pre-eclampsia, and none have systematically assessed the potential of candidates. A 2020 narrative review of proposed candidate medicines for the prevention and treatment of pre-eclampsia included some of the high-potential candidates included in this study, such as gefitinib, etanercept and rhPlGF^96^. Due to the large number of articles identified using the descriptive review search strategy, the authors only focussed on studies published from 2015^96^. This means that inactive (though potentially promising) candidates with studies before 2015 are excluded. Published articles were assessed subjectively and prioritised drugs with in vivo preclinical data, to the exclusion of in vitro data^96^. Therefore, the review did not comprehensively assess all available preclinical data. In the current study, the process of assessing and ranking candidates against the prioritisation matrix provides a more objective, drug-agnostic method of ranking candidate potential. It has thus identified several high-potential candidates that were not included in previous reviews, namely Mze786, cyclosporin A, SynB1-ELP-p50i, puerarin, mangiferin, l-ergothioneine, azathioprine and sufentanil.

Across the pipeline, the most common proposed mechanism of action was a reduction in sFlt-1 production or inhibition of its release, either directly or through surrogate targets and signalling pathways^97,98^. sFlt-1 is correlated with the severity of pre-eclampsia, and is increasingly used to facilitate diagnosis of pre-eclampsia, making it a promising target for therapeutic intervention^99^. Evidence from human trials shows that removing excess sFlt-1, via therapeutic apheresis with a plasma-specific dextran sulphate column, can reduce circulating levels of this anti-angiogenic protein and safely prolong pregnancies in mothers with preterm pre-eclampsia^94,95^. However, sFlt-1 mediated pre-eclampsia is likely only a subset of the disease. Specifically, late-onset or term pre-eclampsia, which makes up most of the cases of pre-eclampsia globally, appears not to be mediated by angiogenic factors such as sFlt-1^100,101^. As the understanding of sub-categories of pre-eclampsia expands, so too should the preclinical drug targets being explored, to ensure innovation for all types of pre-eclampsia. Greater diversity in target mechanism of action would broaden the scope of possible successful therapies for pre-eclampsia/eclampsia^1,15^.

One of the limitations of the current preclinical pipeline is the lack of diversity of animal models for pre-eclampsia. Our analysis identified that over a third of studies were using experimental models that were inappropriate for investigating the effect of candidates against pre-eclampsia. The RUPP rat model was commonly used for in vivo preclinical pre-eclampsia studies. The candidates tested using this model were often proposed to prevent or treat pre-eclampsia by reducing sFlt-1 signalling. However, this model does not mimic compromised placentation characteristic of sFlt-1-mediated preterm pre-eclampsia, and so this model is better suited for studying term pre-eclampsia^102^. A 2022 review of available animal models for pre-eclampsia identified the Asb4 deletion mouse as useful for studying very early stages of pre-eclampsia and the Dahl salt-sensitive rat model appropriate for investigating superimposed pre-eclampsia^103^. However, neither model was used in any of the preclinical studies we analysed. Future in vivo research may benefit from greater use of non‑human primate models, which appeared in only one preclinical study in our analysis^72^. Western Sydney University and the Ingham Institute have developed a baboon model using uterine artery ligation that more closely replicates the manifestation of pre‑eclampsia in humans^72^. Development of new animal models of pre-eclampsia, or improved tools such as physiologically based pharmacokinetics, or organs on a chip, that closely mimic the aetiologies underlying either preterm or term pre-eclampsia will aid in the development of more targeted NCEs for pre-eclampsia^104,105^.

The prioritisation matrix provides a systematic method to assess the potential of preclinical candidates from discovery to implementation. It provides a robust and systematic methodology that can be applied and adapted to other pregnancy-related conditions, including those in the Maternal Health Pipeline. A limitation of the study revolves around data availability. The analysis relies heavily on publicly available information. NCEs in very early development typically lack data for implementability questions. This is indicative of the lack of end-to-end thinking in drug development for maternal medicines^106^. Repurposed candidates were more likely to score higher in this domain, which reflects the real-world situation where drugs already on the market for other conditions have more information available and move through to clinical trials more rapidly^17^. Candidates being developed by pharmaceutical companies are more likely to have associated commercial intellectual property rights, meaning some information may not be publicly available. As this analysis is considered a living document, the intention is to update it as more evidence is generated, and new candidates emerge.

## Conclusions

We identified eleven candidate medicines at the preclinical stage with high potential for further development. The results from this study can be used to better direct research funding towards these promising candidates. Key limitations in the evidence base on preclinical science for pre-eclampsia medicines include a lack of diversity in target mechanisms of action, and a lack of appropriate animal models for pre-eclampsia. To assist in finding pre-eclampsia-specific medicines, further research into the aetiology of pre-eclampsia is also required. Despite many promising new candidates, R&D in pregnancy-related conditions remains fragile due to limited investment, hindering development of NCEs as potential pre-eclampsia therapeutics. Greater commitment and coordination between industry, academic institutions and the maternal health field are needed to avoid attrition and develop new, better medicines for pre-eclampsia.

### Ethics approval

This study did not include direct human research and therefore we did not seek ethics approval from any institutional review board or ethical body for the execution of this study. No consent from patients was required, as any information relating to pharmaceutical drug use in patients was publicly available and de-identified information from published clinical reporting.

## Supplementary information

Transparent Peer Review file

Supplementary information

Description of Additional Supplementary Files

Supplementary Data 1

Supplementary Data 2

## Data Availability

The database generated and analysed during the current study (2026 version) is publicly available at: https://pipeline.impactglobalhealth.org at the time of publication. All numerical data underlying the figures are provided in the Supplementary Data files.
