# Supplementary material for: The global preclinical research and development landscape for pre-eclampsia and eclampsia therapies
Source: Commun Med (Lond). 2026 Jul 14;6:576. doi: 10.1038/s43856-026-01784-3 (PMC13737145; doi:10.1038/s43856-026-01784-3)
Supplement: Supplementary file 1 [file 43856_2026_1784_MOESM1_ESM.pdf]

nature portfolio

# Peer Review File

# The Global Preclinical Research and Development Landscape for Pre-Eclampsia and Eclampsia Therapies.

Corresponding Author: Ms Jessica Postregna

**This file contains all reviewer reports in order by version, followed by all author rebuttals in order by version.**

Version 0:

Reviewer comments:

Reviewer #1

(Remarks to the Author)
1. Some key drugs that are being tested for preeclampsia in humans are misssing from table 2.

a. Kaneka pharmaceuticals with apheresis (PMID: 26405111 and 21810665)
b. Comanche biopharma with sFlt1 siRNA (PMID: 35847173; 30451990)

2. Animal models section could be expanded to include baboon model of preeclampsia pioneered by the Syndey group.

3. Regulatory challenges for developing a drug in preeclampsia could be expanded

4. Target product profile for pre-term and term preeclampsia could be included

Reviewer #2

(Remarks to the Author)
Dear Authors,

Thank you for the opportunity to review this well written manuscript assessing the preclinical drug development of therapeutics for preeclampsia. The methods for this manuscript are excellent

Major comments:
The search seems to have been done before October 2021. This is more than 4 years ago. In the last 4 years there have been a number of new developments in potential preeclampsia therapeutics.
I would strongly recommend reviewing the literature as the current search is out of date and excludes a number of new and important developments.

Minor comments:
The most recent Cochrane review does not support the use of calcium for preventing preeclampsia (Line 50). I think the WHO is updating their guidelines so the authors should consider revising this to reflect the most recent literature.
A subcutaneous injection is a relatively easy way of administering a drug. Perhaps it should be scored as a 3 (not 2)

Kind regards
Cathy Cluver

Version 1:

Reviewer comments:

Reviewer #2

(Remarks to the Author)
Thank you for the opportunity to review the edited version of this manuscript.

You have excluded any compounds that are planned for clinical trials.
I think this is very misleading and biases the review.

Examples of novel drugs that are not included (after doing a quick internet search) are:
1.siRNA drugs being developed by Comanche (https://comanchebiopharma.com/) which were not yet in clinical trials in 2025
2. DM 199 being developed by (https://www.diamedica.com/pipeline/Preeclampsia) which started clinical trials in 2025
3. Monocolonal antibodies being devloped by Beech biotech (https://www.swissbiotech.org/news/beech-biotech-awarded-1-9-million-grant-to-advance-novel-therapeutic-approach-for-preeclampsia-toward-clinical-development/)
4. sFlt binding therapies produced by Avilar therapeutics (https://avilar-tx.com/pipeline-programs/preeclampsia-and-sflt1/)

For this review to be of clinical relevance I think it would be important to include these and other novel therapeutics, especially if clinical trials are planned.
For a number of these therapeutics, trials are planned but are not executed.
I would also encourage you to reach out to the biotech companies for more information on the novel therapeutics.

It may be very useful to the reader to clearly list (perhaps in a table) the therapeutics that are currently being tested that you found in your search and excluded as they are currently being tested.

Version 2:

Reviewer comments:

Reviewer #2

(Remarks to the Author)
Thank you for addressing my comments.

**Open Access** This Peer Review File is licensed under a Creative Commons Attribution 4.0 International License, which permits use, sharing, adaptation, distribution and reproduction in any medium or format, as long as you give appropriate credit to the original author(s) and the source, provide a link to the Creative Commons license, and indicate if changes were made.
In cases where reviewers are anonymous, credit should be given to 'Anonymous Referee' and the source.
The images or other third party material in this Peer Review File are included in the article's Creative Commons license, unless indicated otherwise in a credit line to the material. If material is not included in the article's Creative Commons license and your intended use is not permitted by statutory regulation or exceeds the permitted use, you will need to obtain permission directly from the copyright holder.
To view a copy of this license, visit https://creativecommons.org/licenses/by/4.0/

Manuscript tracker number: COMMSMED-25-2003-T

Title: The Global Preclinical Research and Development Landscape for Pre-Eclampsia and Eclampsia Therapies.

| Comment | Response | Line/s |
|---|---|---|
| Reviewer #1 | | |
| 1. Some key drugs that are being tested for preeclampsia in humans are missing from table 2. | We thank the reviewer for this helpful comment. We acknowledge that several key drug candidates currently under evaluation in human clinical trials for preeclampsia are not included in Table 2. This omission is intentional, as Table 2 is specifically focused on therapeutic candidates that remain in the preclinical stage of development. Drugs already being tested in clinical (human) trials fall outside the scope of this table but are discussed elsewhere in the manuscript. | N/A |
| a. Kaneka pharmaceuticals with apheresis (PMID: 26405111 and 21810665) | Apheresis, being explored by Kaneka pharmaceuticals, was not included in our analysis because the maternal health pipeline evaluated in this study was limited to therapeutic products (drugs, biologics, and dietary supplements). As a medical procedure rather than a medicinal product, apheresis fell outside the preclinical pipeline assessment. Nevertheless, we recognise its relevance in the field and have highlighted in the Discussion sections that non-pharmacological interventions, including apheresis, are also being investigated for the management of pre-eclampsia. | Lines 408 - 411and 438 - 441 |
| b. Comanche biopharma with sFlt1 siRNA (PMID: 35847173; 30451990) | We thank the reviewer for highlighting CBP-4888 (the sFlt-1–targeting siRNA developed by Comanche Biopharma) as a promising therapeutic candidate. We acknowledge its relevance to the evolving treatment landscape for pre-eclampsia. This candidate was not included in our analysis because it has been classified as being in Phase I clinical trials. As our review focused specifically on candidates within the *preclinical* pipeline, CBP-4888 fell outside the | Lines 398 - 402 |

| | | |
|---|---|---|
| | predetermined scope of our analysis.<br>However, we recognize its relevance as a novel, specifically developed candidate for pre-eclampsia treatment and so this has been discussed in the Discussion. | |
| 2. Animal models section could be expanded to include baboon model of preeclampsia pioneered by the Syndey group. | We have expanded the animal models' section in the discussion to include the baboon model of pre-eclampsia. | Lines 460 - 463 |
| 3. Regulatory challenges for developing a drug in preeclampsia could be expanded | We thank the reviewer for the suggestion. We have incorporated a sentence into the manuscript acknowledging prominent regulatory barriers including limited industry engagement, reliance on off-label medication use, and hesitancy to conduct clinical trials in pregnant populations. | Lines 406 - 408 |
| 4. Target product profile for pre-term and term preeclampsia could be included | We appreciate the reviewer's suggestion. Target Product Profiles have already been published and adopted by the World Health Organisation. We have now noted this in the manuscript and added the appropriate citations. | Lines 88 – 90 |
| Reviewer #2 | | |
| Major comments | | |
| The search seems to have been done before October 2021. This is more than 4 years ago. In the last 4 years there have been a number of new developments in potential preeclampsia therapeutics.<br>I would strongly recommend reviewing the literature as the current search is out of date and excludes a number of new and important developments. | Thank you for this helpful feedback. As the maternal health pipeline has now been updated to October 2025, our paper now includes an updated analysis of the preclinical pipeline. This includes any new changes to existing candidates as well as the addition of new candidates since 2021. | Throughout |
| Minor comments | | |
| The most recent Cochrane review does not support the use of calcium for preventing preeclampsia (Line 50). I think the WHO is updating their guidelines so the authors should consider revising this to reflect the most recent literature. | We have revised the introduction to include the results from the most recent Cochrane review that does not support the use of calcium in preventing pre-eclampsia. | Lines 76 - 78 |
| A subcutaneous injection is a relatively easy way of administering a | We agree that a subcutaneous injection is easier to administer than intramuscular injection. As such, we have changed the scoring for | Supplementary Table 1 |

| | | |
|---|---|---|
| drug. Perhaps it should be scored as a 3 (not 2) | subcutaneous injection from a 2 to a 3. | |

Manuscript tracker number: COMMSMED-25-2003B

Title: The Global Preclinical Research and Development Landscape for Pre-Eclampsia and Eclampsia Therapies.

| Comment | Response | Line/s |
|---|---|---|
| Reviewer #1 | | |
| You have excluded any compounds that are planned for clinical trials.<br>I think this is very misleading and biases the review.<br><br>Examples of novel drugs that are not included (after doing a quick internet search) are:<br>1.siRNA drugs being developed by Comanche (https://comanchebiopharma.com/) which were not yet in clinical trials in 2025<br>2. DM 199 being developed by (https://www.diamedica.com/pipeline/Preeclampsia) which started clinical trials in 2025<br>3. Monocolonal antibodies being devloped by Beech biotech (https://www.swissbiotech.org/news/beech-biotech-awarded-1-9-million-grant-to-advance-novel-therapeutic-approach-for-preeclampsia-toward-clinical-development/)<br>4. sFlt binding therapies produced by Avilar therapeutics (https://avilar-tx.com/pipeline-programs/preeclampsia-and-sflt1/)<br><br>For this review to be of clinical relevance I think it would be important to include these and other novel therapeutics, especially if clinical trials are planned.<br>It may be very useful to the reader to clearly list (perhaps in a table) the therapeutics that are currently being tested that you found in your search and excluded as they are currently being tested. | We thank the reviewer for this valuable comment. We recognize the importance of novel candidates that are planned for or currently in clinical trials. In response, we have added a new section to the Results describing novel candidates with planned or early clinical trials. This includes the candidates highlighted by the reviewer, as well as three additional candidates that were identified in the Maternal Health Pipeline. We have also included a summary table, as suggested, and updated the methods to reflect this change. | Lines 167-169, 394-403, 414-418 and Table 3. |
