# Supplementary material for: The global preclinical research and development landscape for pre-eclampsia and eclampsia therapies
Source: Commun Med (Lond). 2026 Jul 14;6:576. doi: 10.1038/s43856-026-01784-3 (PMC13737145; doi:10.1038/s43856-026-01784-3)
Supplement: Supplementary file 2 [file 43856_2026_1784_MOESM2_ESM.pdf]

Supplementary Information

Supplementary Table 1. Prioritisation Matrix: Scoring for assessment of preclinical candidate medicines for pre-eclampsia.

a) Quality of evidence for preclinical candidate medicines for pre-eclampsia. Calculation of candidate quality of evidence is first calculated per study. For each variable, the study is assigned a coding value. These coding values are then added together to obtain the overall quality of evidence for that study (max total coding value of 48 per study). For candidates with multiple studies, the average score among all associated studies will then be calculated. Once this score has been determined, the final coding value (regarding number of studies per candidate) can be added to this average score. This creates the final quality of evidence score given to the preclinical drug candidates' (max total coding value of 53 per candidate).

Variable	Answer options	Answer criteria	Coding value
Clearly stated aims/objectives	Adequately scored	Well defined and specific aims and objectives to the study.	4
	Inadequately scored	Hypothesis stated but no set aim mentioned beyond testing the drug (vague aims).	2
	Not specified	No aims, hypothesis and/or objectives mentioned.	0
Detailed explanation of sample size calculation	Adequately scored	Well defined sample size calculation: all calculations and components reported; sample size stated.	4
	Inadequately scored	Sample size stated, but no calculation present.	2
	Not specified	No mention of sample size or calculation.	0
	Adequately scored	*For animal model:*	4

Detailed explanation of sampling technique		Pre-eclampsia animal model used.<br>*For in vitro model:*<br>Pre-eclampsia *in vitro* model used; cells taken from primary human tissue either from a patient with pre-eclampsia or cells are from a normal pregnancy and then placed under pre-eclampsia-like conditions (eg: hypoxia).	
	Inadequately scored	*For animal model:*<br>Pregnant animal model used.<br>*For in vitro model:*<br>Cells taken from primary human tissue from a patient with normal pregnancy.	2
	Not specified	No information on population; non-pregnant model used.	0
Details of comparison group	Adequately scored	Vehicle control group mentioned, and details provided.	4
	Inadequately scored	Mention of comparison group with insufficient detail.	2
	Not specified	No mention of comparison group.	0
Detailed explanation of methodology	Adequately scored	Easy to comprehend procedure, explanation of terms and techniques.	4
	Inadequately scored	Somewhat vague procedure but can be followed appropriately or detailed but hard to follow, no missing information.	2
	Not specified	Confusing/vague procedures, terms/techniques not expanded upon, missing information/procedures.	0

Operator details	Adequately scored	Number of operators and training details identified.	4
	Inadequately scored	Number of operators or training details identified – one of the two.	2
	Not specified	Neither identified.	0
Randomisation	Adequately scored	Sequence generation and allocation concealment reported.	4
	Inadequately scored	Randomisation mentioned but not elaborated.	2
	Not specified	No mention of randomisation.	0
Method of outcome measurement	Adequately scored	Measurement of outcome/experiment performed has a clear rationale.	4
	Inadequately scored	Measurement of outcome/ experiment performed is mentioned but has a vague/no rationale.	2
	Not specified	Measurement of outcome is not mentioned.	0
Outcome assessor details	Adequately scored	Number of assessors and training details identified.	4
	Inadequately scored	Number of assessors or training details identified – one of the two.	2
	Not specified	Neither identified.	0
Blinding	Adequately scored	Blinding of operator, assessor and statistician maintained throughout study clearly specified and maintained throughout study.	4
	Inadequately scored	Blinding is mentioned but not elaborated.	2
	Not specified	No mention of blinding.	0
Statistical analysis	Adequately scored	Correct statistical analysis is performed and software package is mentioned.	4

	Inadequately scored	Correct statistical analysis is performed but no mention of software package.	2
	Not specified	No mention of statistical analysis or software package; wrong statistical analysis used.	0
Presentation of results	Adequately scored	Results/data from all experiments have been presented in a clear and concise manner either visually or in writing.	4
	Inadequately scored	Results/data from all experiments have been reported in a somewhat vague or overly complicated manner.	2
	Not specified	No mention of results or some results/data from experiments performed is missing.	0
Calculate average score from all associated studies for that drug candidate, then add coding value for next question.			
Number of studies per candidate	Five	Five studies performed on drug candidate.	5
	Four	Four studies performed on drug candidate.	4
	Three	Three studies performed on drug candidate.	3
	Two	Two studies performed on drug candidate.	2
	One	One study performed on drug candidate.	1

b) Product development for preclinical candidate medicines for pre-eclampsia.

Calculation of candidate product development is calculated per candidate. For each variable, the candidate is assigned a coding value. These coding values are then added together to obtain the overall candidate product development (max total coding value of 26).

Variable	Answer options	Answer criteria	Coding value
TRL1 – basic scientific research			
Have basic principles about the drug candidate been observed and reported?	Yes	Basic principles observed, published competitive landscape and market analysis report.	2
	Probably yes	Basic principles observed, drug being developed by pharma company.	1
	Probably no	Basic principles observed but no competitive landscape and market analysis report.	0
	No	No basic principles observed.	-1
	Unsure	Unknown or basic principles observed, and drug is being developed by an academic institute.	0
TRL2 – concept formulation			
Has a drug concept been formulated (development of hypothesis and experimental designs) and has a clear product profile been defined?	Yes	Drug concept formulated and product profile defined for a prevention or treatment of pre-eclampsia.	2
	To an extent	Drug concept formulated and product profile defined for a symptom management of pre-eclampsia.	1
	No	No drug concept formulated, and no product profile defined.	-1
	Unsure	Unknown.	0
TRL3/3.5 – mechanism of action			
Has efficacy been demonstrated in an	Yes	Strong evidence provided by the *in vitro* studies performed, demonstrate that the drug can be	2

in vitro model for pre-eclampsia?		used to prevent/treat pre-eclampsia.	
	To an extent	*In vitro* studies have provided some evidence that the drug works to prevent/treat pre-eclampsia, but they mention that future studies are needed to confirm this evidence.	1
	No	No evidence of efficacy in an *in vitro* model.	0
	Unsure	Unknown.	0
Has efficacy been demonstrated in an animal model for pre-eclampsia?	Yes	Strong evidence provided by the animal studies performed, demonstrate that the drug can be used to prevent/treat pre-eclampsia.	2
	To an extent	Animal studies have provided some evidence that the drug works to prevent/treat pre-eclampsia, but they mention that future studies are needed to confirm this evidence.	1
	No	No evidence of efficacy in an animal model.	0
	Unsure	Unknown.	0
Is the mechanism of action for the drug candidate known?	Yes	Repurposed drug. All of the following is known: target, pathway, or end-result/indication.	3
	Probably yes	Two of the following is known: target, pathway, or end-result/indication.	2
	Probably no	One of the following is known: target, pathway, or end-result/indication.	1
	No	Mechanism of action has not yet been discovered.	-1
	Unsure	Unknown.	0
Is there a proposed mechanism of action for how the drug	Yes	Drug is suspected to work via “this mechanism of action” against pre-eclampsia.	2

candidate acts against pre-eclampsia?	No	Drug seems to work against pre-eclampsia however it is unknown how it does so.	0
	Unsure	Unknown	0
Is the mechanism of action for how the drug candidate acts against pre-eclampsia evidenced in a pre-eclampsia model?	Yes	Drug works via “this mechanism of action” as evidenced in a pre-eclampsia model. (If yes, candidate is a TRL3.5)	2
	No	Suspected mechanism of action has not been evidenced in a pre-eclampsia model.	0
	Unsure	Unknown.	0
Does the drug candidate improve pre-eclampsia outcomes?	Yes	Drug candidate has been shown to improve pre-eclampsia outcomes in an animal pre-eclampsia model.	2
	To an extent	Drug candidate has been shown to improve pre-eclampsia outcomes in an *in vitro* pre-eclampsia model OR it only improves pre-eclampsia outcomes as symptom management OR drug has not been tested for fetal outcomes only maternal outcomes.	1
	No	Either: 1- drug candidate has not shown improvements to pre-eclampsia outcomes in pre-eclampsia models OR<br>2. pre-eclampsia outcomes were not assessed in a pre-eclampsia model.	-1
	Unsure	Unknown.	0
TRL4 – toxicology and safety			
Has the drug demonstrated any safety concerns in pregnant and lactating women?	Yes	Safety concerns have been raised in a clinical safety trial or in a large retrospective cohort study in pregnant or lactating women.	-2
	To an extent	Safety concerns have been raised in one or two small retrospective cohort studies in pregnant or	-1

		lactating women OR safety concerns have been raised in preclinical studies.	
	No	No safety concerns in pregnant or lactating women.	1
	Unsure	Unknown.	0
Has a safe allowable human dose been established for the drug candidate?	Yes	Repurposed drug: safe dosage for humans has been approved for drug by FDA.	2
	To an extent	A safe dose for humans has been approved for NCE candidates.	1
	No	No safe human dose had been established for this candidate.	0
	Unsure	Unknown.	0
Was an equivalent safe human dosage used in the study?	Yes	An acceptable safe human dosage was used in the studies.	1
	No	A safe human dosage was not used in the studies; dosage was higher than safe allowable dose used to demonstrate efficacy.	-2
	Unsure	Either unknown or safe allowable human dose has not been established.	0
TRL5 – Chemistry, Manufacturing and Control (CMC) & Absorption, Distribution, Metabolism and Excretion (ADME)			
Have formulation studies been performed for the drug candidate?	Yes	Repurposed drug. NCE candidates have mentioned that formulation studies have been published.	1
	No	Explicit mention that no formulation studies have been performed.	0
	Unsure	Unknown if formulation studies have been performed/no mention of formulation studies.	0
Is controlled production of the drug candidate performed under cGMP (current Good	Yes	Repurposed drug. Evidence needed for NCE candidates to ensure that the candidate is manufactured under cGMP.	1
	No	Not manufactured under cGMP.	0
	Unsure	Unknown.	0

Manufacturing Practices)?			
Has a half-life been established for the drug candidate?	Yes	Half-life established.	1
	No	Half-life not established.	0
	Unsure	Unknown.	0
Is the metabolic pathway of the drug candidate known?	Yes	Metabolic pathway known.	1
	No	No research into metabolic pathway of the drug.	0
	Unsure	Unknown.	0
Are any potential drug-drug interactions known?	Yes	Proven interaction interactions with other drugs.	-2
	To an extent	Potential/unproven interaction with another drug.	-1
	No	No interactions with other drugs.	1
	Unsure	Unknown.	0

c) Implementability for preclinical candidate medicines for pre-eclampsia. Calculation of candidate implementability is calculated per candidate. For each variable, the candidate is assigned a coding value. These coding values are then added together to obtain the overall implementability (max total coding value of 11).

Variable	Answer options	Answer criteria	Coding value
Therapeutic indication	Prevent underlying aetiology	N/A	4
	Treat underlying aetiology	N/A	3
	Prevent and treat	N/A	4
	Symptom management	N/A	1
	Unsure	Unknown.	0
Route of administration	Intraarterial	N/A	1
	Intravenous	N/A	1
	Subcutaneous	N/A	3
	Oral	N/A	4
	Intraperitoneal	N/A	1
	Intramuscular	N/A	2
	Unspecified	Unknown.	0
Storage conditions – is cold chain required?	Yes	Storage/transport of drug within the range of +2°C to +8°C (cold chain).	-2
	No	Cold chain not required.	2
	Unsure	Unknown.	0
Place of development	Academic institute	N/A	1
	Private company	N/A	1
	Both	N/A	1
	Unsure	Unknown.	0

d) Threshold for ranking of potential for preclinical candidate medicines for pre-eclampsia. The summation of all coding values from the three themes are used to determine the final ranking of potential for each candidate. Quality of evidence accounts for 58.89% (max total coding value of 53), product development accounts for 28.89% (max total coding value of 26), and implementability accounts for 12.22% (max total coding value of 11) of the final score given to the preclinical candidates assessed. The maximum score a candidate can achieve is 90. The cut-off points as shown below were sense checked with experts in the field. The cut-off points and associated rankings allows candidates that are considered high potential the systematic assessment done with the prioritisation assessment matrix to be scored as high.

Final ranking	Preclinical cut-off points
High potential	>55
Medium potential	41-54
Low potential	<40

Supplementary Table 2. Excluded candidates.

Candidate	Reason for Exclusion
Angiotensin receptor blocking antibody fragments	Data not publicly available; private company researching under commercial intellectual property.
Anti-MBG human monoclonal antibody	Data not publicly available; private company researching under commercial intellectual property.
B7-33	Study was not a full, published preclinical study; abstract only.
C103	Data not publicly available; private company researching under commercial intellectual property.
Dasatinib	Studies report adverse effects of the drug candidate.
EG 101	Data not publicly available; private company researching under commercial intellectual property.
GMA 312	Data not publicly available; private company researching under commercial intellectual property.
GPCR-AAB binding aptamers	Theoretical/hypothesised drug candidate.
Gynaecological disorder therapeutics	Data not publicly available; private company researching under commercial intellectual property.
Handle-region peptides (HRP)	Data not publicly available; private company researching under commercial intellectual property.
Kynurenine	Data not publicly available; private company researching under commercial intellectual property.
Magnocaine	Theoretical/hypothesised drug candidate.
Menadione	Studies report adverse effects of the drug candidate.
MitoQ	Studies report adverse effects of the drug candidate.
Mycophenolate mofetil	Studies report adverse effects of the drug candidate.
Nicorandil	Data not publicly available; private company researching under commercial intellectual property.
PN 101 RS	Data not publicly available; private company researching under commercial intellectual property.
Prasugrel	Study was not a full, published preclinical study; abstract only.

Propolis	Study was not a full, published preclinical study; abstract only.
Simvastatin	Studies report adverse effects of the drug candidate.
Tocotrienol	Study was not a full, published preclinical study; abstract only.
Trehalose	Study was not a full, published preclinical study; abstract only.
VEGF-B	Studies report adverse effects of the drug candidate.

Supplementary Text 1. Medium and low potential candidates.

Medium potential candidates

Of the 83 candidates analysed, 63 were identified as medium potential having a final score between 41 and 54 (Supplementary Data 1). These candidates tend to score lower in product development due to insufficient safety and efficacy studies (Supplementary Data 1). These candidates tend to also score lower in implementability due to route of administration and cold chain requirements (Supplementary Data 1). Five (8.0%) of these candidates are being investigated by pharmaceutical companies and 52 (82.5%) by academic institutes however the remaining six (9.5%) are being explored by both (Supplementary Data 2). Three (4.8%) medium potential candidates are being investigated for pre-eclampsia prevention, 39 (61.9%) candidates are being explored for treatment, and 10 (15.9%) candidates are for symptom management (Supplementary Data 2). The remaining 11 (17.5%) candidates are being investigated for both the prevention and treatment of pre-eclampsia (Supplementary Data 2). The majority of medium potential candidates are at a TRL 3 and TRL 3.5 meaning they need further research into the efficacy of the candidates (Supplementary Data 1, Supplementary Data 2).

Medium potential candidates at a TRL 4

Some medium potential candidates performed well enough in efficacy and mechanism of action preclinical studies to warrant being at a TRL 4 (GYY4137, pyridostigmine, sFlt-1-targeting small interfering RNA (siRNA) and tetramethylpyrazine; Supplementary Data 1).[39,40,57,80-84] GYY4137 is a slow releasing H^2S-releasing drug that has the ability to release H^2S in a slow and sustained manner more similar to endogenous production of H^2S.[21] This NCE was being investigated by Sao Paulo State University (Brazil) for the treatment of pre-eclampsia targeting HO-1. Two preclinical studies were produced in the DOCA salt rat and DL-propargylglycine mouse model (Supplementary Data 1).[80,81] GYY4137 can deliver H^2S without potential toxicity, unlike fast-release sodium H^2S which delivers H^2S as a single concentrated burst.[80] GYY4137 was more advantageous to restore systolic blood pressure and protect against uteroplacental fetal growth restriction and oxidative stress.[80]

Pyridostigmine, another NCE at TRL 4, is a repurposed drug that functions as an acetylcholinesterase inhibitor to indirectly inhibit the TNFα signalling pathway (Supplementary Data 1).[21] Xi'an Jiaotong University (China) was investigating this drug as a pre-eclampsia treatment in RUPP rats and pre-eclamptic placental samples.[57] This study showed that pyridostigmine reduced MAP, placental oxidative stress, apoptosis, and inflammation in RUPP rats, and restored TNF-α–reduced cell viability *in vitro*.[57]

Another medium potential candidate at a TRL 4 is sFlt-1-targeting siRNA which utilises placenta-specific nanoparticle delivery.[21] This candidate was being investigated by academic institutes in China for the treatment of pre-eclampsia and has scored high in efficacy and mechanism of action studies resulting in a TRL 4 score (Supplementary Data 1). Two preclinical studies have been performed for this candidate, both *in vitro* and *in vivo* models induced with TNFα, finding significantly decreased sFlt-1 levels and improved pre-eclampsia outcomes without a toxic effect when administered with this siRNA.[82,83]

Tetramethylpyrazine is at TRL 4 (Supplementary Data 1). It is a traditional Chinese medicine that has been used extensively for cardiovascular and cerebrovascular diseases.[21] This repurposed drug candidate was being investigated by academic institutes for the treatment of pre-eclampsia targeting CHOP and GRP78 miRNAs (Supplementary Data 2). Two preclinical studies have been performed on these candidates both in *in vitro* and *in vivo* pre-eclamptic models including both the L-NAME and LPS rat models.[38,39] They found that treatment with tetramethylpyrazine caused the inhibition of CHOP and GRP78 miRNA which resulted in downstream effects on increasing NO synthesis leading to improved placental and fetal weights.[38,39]

Despite being at a TRL 4, three of these medium potential candidates scored low in implementability (Supplementary Data 1). GYY4137 and tetramethylpyrazine are both administered intravenously, and sFlt-1-targeting siRNA is administered subcutaneously or intramuscularly. Consequentially, these three candidates require cold chain to be transported and stored stably (Supplementary Data 1). Pyridostigmine scored higher in implementability as it is administered orally without cold chain requirements.

The remaining 59 medium potential candidates scored less than a TRL 4, due to lower scores in preclinical efficacy studies. Although some of these candidates may have scored higher than those at TRL 4, either the mechanism of action or efficacy of the candidate was not explored sufficiently, which has prevented them from progressing to a higher TRL. These candidates are therefore either at a TRL 3 or TRL 3.5.

Low potential candidates

Of the 83 candidates analysed, nine were identified as low potential having a final score of 40 or below (Supplementary Data 1). These candidates tend to score lower in quality of evidence and product development. Six (66.7%) of these candidates are being investigated by academic institutes and the three remaining (33.3%) are being explored by both academic institutes and pharmaceutical companies (Supplementary Data 2). One (11.1%) candidate is being investigated for pre-eclampsia prevention, five (55.5%) are being explored for treatment, and three (33.3%) candidates are for symptom management (Supplementary Data 2). Of the 12 low potential candidates, two (22.2%) used a dosage in preclinical studies that was up to 25 times higher than the safe human dosage (Supplementary Data 1). All of the low potential candidates are at a TRL 3 meaning they need further research into the efficacy and mechanism of action of the candidates (Supplementary Data 1).
