# Supplementary material for: The global preclinical research and development landscape for pre-eclampsia and eclampsia therapies
Source: Commun Med (Lond). 2026 Jul 14;6:576. doi: 10.1038/s43856-026-01784-3 (PMC13737145; doi:10.1038/s43856-026-01784-3)
Supplement: Supplementary file 3 [file 43856_2026_1784_MOESM3_ESM.pdf]

Description of Additional Supplementary Files

File name: Supplementary Data 1

Description: Excel spreadsheet with analysis (raw data).

File name: Supplementary Data 2

Description: Extended summary of preclinical candidate medicines for preeclampsia/eclampsia.
